# Evaluation of Relationship between Body Mass Index with *Vitamin D Receptor* Gene Expression and Vitamin D Levels of Follicular Fluid in Overweight Patients with Polycystic Ovary Syndrome

**DOI:** 10.22074/ijfs.2017.4704

**Published:** 2017-02-16

**Authors:** Esmat Aghadavod, Hakimeh Mollaei, Mohammad Nouri, Hamed Hamishehkar

**Affiliations:** 1 Gametogenesis Research Center, Kashan University of Medical Sciences, Kashan, Iran; 2 Research Center for Pharmaceutical Nanotechnology, Tabriz University of Medical Sciences, Tabriz, Iran; 3 Department of Biology, Science and Research Branch of Islamic Azad University, Tehran, Iran; 4 Women Reproductive Health Research Center, Tabriz University of Medical Sciences, Tabriz, Iran; 5 Drug Applied Research Center, Tabriz University of Medical Sciences, Tabriz, Iran

**Keywords:** Polycystic Ovary Syndrome, Vitamin D, Granulosa Cells, *Vitamin D Receptor*, Follicular Fluid

## Abstract

**Background:**

Polycystic ovary syndrome (PCOS) is the most common endocrine disorder associated with reproductive disorders and metabolic dysfunctions including insulin resistance. The roles of vitamin D in the regulation of metabolic modulations specifically involving insulin and reproduction processing are introduced. In addition, obesity appears to be closely associated with severity of PCOS. The present study is to evaluate the effect of body mass index (BMI) on vitamin D levels in follicular fluid and *vitamin D receptor (VDR)* expression levels in granulos cells.

**Materials and Methods:**

A comparative study was conducted on 80 women with average age of 20-35 years referred for *in vitro* fertilization (IVF). Patients were divided into four groups, and serum levels of testosterone and insulin resistance (IR) were evaluated at the puncture time. Also, vitamin D levels of follicular fluid were evaluated. *VDR* gene expression was assayed by quantified-polymerase chain reaction (PCR) technique. Correlations were evaluated with calculation of the Spearman coefficient, and also independent relationships were assessed by means of multiple regression analysis.

**Results:**

Vitamin D levels of follicular fluid decreased in PCOS patients compared with non-PCOS. Also, over-weight individuals had lower vitamin D levels compared with normal-weight patients. Vitamin D levels of follicular fluid were highly correlated with BMI (r=-0.51, P<0.01). Homeostatic model assessment-IR (HOMA-IR) values were significantly higher in women of PCOS/overweight and PCOS/normal weight in comparison with women of non-PCOS/normal weight (P<0.01). The gene expression data of *VDR* in granulosa cells were significantly lower in the PCOS/overweight group compared with the non-PCOS/normal weight (P<0.01).

**Conclusion:**

The findings indicated significant differences in *VDR* gene expression in granulosa cells and vitamin D of follicular fluid in PCOS/overweight patients.

## Introduction

Polycystic ovary syndrome (PCOS) is the most common endocrine disorder that can affect 8% to 10% of women in their reproductive age (1, 2). PCOS is associated not only with reproductive disorders, but also with significantly increased risks of metabolic dysfunctions, including insulin resistance (IR) (3), dyslipidemia (4), systemic inflammation (5), increased oxidative stress (6), and endothelial dysfunction (7). It can be noticed that PCOS, as a heterogeneous androgen excess disorder with varying degrees of reproductive and metabolic abnormalities, is determined by the interaction of multiple genetic and environmental factors (8).

Several studies have revealed that certain metabolic disturbances such as IR and hyperinsulinemia are majour defects in the majority of PCOS patients (9, 10). Emerging data characterize serious roles for vitamin D in biological processes, including regulation of cellular growth (11), differentiation, and metabolic modulations specifically involving insulin action (12, 13). Among the many physiologic processes influenced by vitamin D, serious roles in reproductive physiology are submitted (14). Biological actions of vitamin D are intermediated through *vitamin D receptor (VDR)* gene expression which is a member of the steroid/thyroid nuclear hormone receptor superfamily, displayed in calcium-regulating tissues, intestines (12), the skeleton (15), parathyroid glands (16), and reproductive tissues including ovary, uterus, placenta (17), testis (18), and granulosa cells (19, 20).

Additionally, investigations of animals have confirmed the role of calcium in oocyte maturation and its impact on the resumption and progression of follicular development. Furthermore, disturbances in calcium regulation can be responsible for follicular arrest (21). Previous study have suggested the functions of vitamin D in reproduction (22). They have also indicated that *VDR* regulates more than 3% of the human genome, including genes that are crucial for glucose metabolism. *VDR* is a transcription factor regulating the transcription of other downstream genes in many tissues that are crucial for glucose metabolism (23, 24). On the other hand, calcium fluxes and regulation of intracellular calcium stores are essential in the regulation of insulin secretion by b-cells. Therefore, vitamin D and *VDR* gene are important factors in calcium regulation and control of bcell functions, respectively. This is further supported by the fact that low vitamin D levels are associated with IR and that they can induce type-2 diabetes in PCOS patients (25). However, the exact mechanisms underlying the association of vitamin D and IR are not fully understood. One complication of PCOS is obesity which appears to be closely associated with severity of the disease phenotype (26).

In Iran, more than half of the patients with PCOS are either overweight or obese (27). It is well known that obesity influences the phenotypic expression of PCOS and might play a significant role in the pathophysiology of hyperandrogenism, severity of insulin resistance, and also chronic anovulation (28). Increased adiposity is associated with several abnormalities of sex steroid metabolism and results in increased androgen production and suppression of sex hormone binding globulin (SHBG) (29). Thus, obesity may affect vitamin D levels in healthy women and PCOS patients. The present study aimed to evaluate the effect of obesity on vitamin D levels in follicular fluid and *VDR* expression levels in granulosa cells. The results of this research may contribute to the diagnosis and treatment of overweight PCOS patients.

## Materials and Methods

The current comparative study was carried out on 80 women of 20 to 35 years old and who referred to Alzahra-Hospital of Tabriz, Iran for *in vitro* fertilization (IVF). Before entering the study, all the patients provided written consent forms. This study was approved by the Ethics Committee of Tabriz University of Medical Sciences (code: 5/4/2781). Weight and height of all the patients were measured, and then the body mass index (BMI, Kg/m^2^) was calculated by dividing weight by height square. The patients were divided into two groups based on BMI categories, using the specified criteria by the World Health Organization (WHO): the normal weight and overweight groups with the BMI of 18.5-24.9 and 25-29.9, respectively. The control group consisted of 40 non-PCOS patients (20 normal weights and 20 over-weights) who had referred for IVF due to tubal and/or male infertility or even ovulatory volunteers with normal ovaries. The PCOS group included 40 patients (20 normal weights and 20 over-weights) who had referred for IVF. These patients were recognized based on Rotterdam Consensus criteria and had at least two of the three following criteria: ovulatory disturbance, hyerandrogenism, and more than twelve 2 to 9 mm follicles in each ovary. The exclusion criteria were as follows: history of menstrual disorders such as cycle length either less than 25 days or more than 35 days, patients with other endocrine disorders or neoplastic causes of hyperandrogenemia such as androgen-secreting tumors (serum testosterone levels above 0.6 ng/mL), congenital adrenal hyperplasia, and Cushingʼs syndrome.

### Ovarian stimulation

For pituitary down-regulation, the patients were treated with 0.5-1 mg subcutaneous (SC) injection of gonadotropin releasing hormone (GnRH) agonist (Lucrin, Abbott Pharmaceuticals, Kurnell, Australia), depending on the age of every woman. When at least three follicles reached a diameter of almost 17 mm and the levels of peripheral plasma estradiol concentrations were ≥3 nmol/l, 5000-IU of human chorionic gonadotropin (hCG, Profasi, Serono, Aubonne, Switzerland) was given as a single IM injection. Thirty six hours after hCG administration, the oocytes were retrieved and collected with sterile Pasteur pipettes, and then the remainder of the follicular fluid was poured into 50 ml sterile falcon tubes for subsequent isolation of granulosa cells. The follicular fluid was centrifuged, and vitamin D levels were measured in the supernatant. 25-OH vitamin D was estimated by chemiluminescence enzyme immunoassay (IDS, Boldon, UK). The blood samples were obtained before the operation for subsequent biochemical analyses. These samples were analyzed for follicle-stimulating hormone (FSH), luteinizing hormone (LH), testosterone, prolactin, insulin, and glucose levels.

### Granulosa cells collection

The freshly collected follicular fluid samples were then centrifuged at 3000 rpm for 1 minute at 4°C; afterwards, 4 ml of phosphate buffer saline (PBS) was added to the pallet. After mixing, the solution was placed on 50% Percoll gradient (Amersham Pharmacia Biotech, Uppsala, Sweden). The sample was centrifuged at 700 rpm for 30 minutes to remove red blood cells. After the centrifugation, the granulosa cells were placed between PBS and Percoll solutions. The cells were harvested by gentle pipetting, and also washed several times with PBS, and used for RNA extraction and cDNA synthesis. The total RNA of the collected samples was the recommended protocol of manufacture. In brief, 1 ml of RNX plus was added to the sample in a clean RNase-free tube. The sample was homogenated via gentle up and downing and then was incubated for 5 minutes at room temperature. After adding chloroform (200 μl), the mixture was incubated at room temperature for 5 minutes and was centrifuged at 12,000 rpm for 15 minutes at 4°C. The aqueous phase containing RNA was transferred to a clean RNase-free tube. This solution was put on ice, and 500 μl of ice isopropanol was added to it; the sample was then incubated for 30 minutes at -80°C. Afterwards, the tube was centrifuged at 12,000 rpm for 15 minutes at 4°C, and the supernatant was discarded. The pellet, including the total RNA, was washed using 75% ethanol and was centrifuged at 7,500 rpm for 8 minutes. After drying ethanol, the RNA pellet was re-suspended in 50 μl or less of DEPC-treatment water. The concentration of total RNA was calculated based on OD 260/280 ratio measurements as a means to address the purity of RNA.

To confirm the integrity of the extracted RNA, it was electrophoresed. The genomic DNA was removed from the extracted RNA by adding RNase free DNase I (Thermo, Fermentase). The cDNA was synthesized with Moloney murine leukemia virus reverse-transcriptase (MMLV-RT, 200 U/μl, Sigma-Aldrich Co., UK) according to the manufacture’s protocol. As soon as the RNA was isolated from the granulosa cells, the reverse transcriptase reactions were performed on all the samples to generate cDNA.

To quantify the mRNA expression levels of *VDR* gene in the granulosa cells, real-time polymerase chain reaction (RT-PCR) was performed on a Bio-Rad iQ5 system (Bio-Rad Laboratories, Hercules, USA), using EVA-Green quantitative PCR mix kit (Sinaclon, Tehran-Iran). *GAPDH* gene was used as reference standard gene for all analyses to control the amount of the synthesized cDNA. PCR reactions were carried out in triplicate for each sample, and then the mean of the three readings was taken as fold-induction value. Fold change (X) values were calculated, using X=2^-ΔΔCt^ equation, in which ΔCt represents the difference between the Ct values of the target genes and the Ct values of the reference standard genes, and ΔΔCt describes the difference between ΔCt value of each sample for each target gene and the average ΔCt of the reference standard gene. The sequence of PCR primers for amplifying *VDR* and *GAPDH* genes is provided in Table 1. IR was estimated, using the homeostatic model assessment-IR (HOMA-IR) method. In addition, HOMA-IR was calculated as the product of the fasting plasma insulin value (mU/mL) and the fasting plasma glucose value (mg/dL). Insulin levels were also estimated by ELISA kit (Siemens, Erlangen, Germany) according to the manufacture’s recommendations.

### Statistical analysis

All statistical procedures were run, using SPSS-16 software (SPSS Inc., Chicago, IL), and P<0.05 was considered statistically significant. Normal distribution of data was evaluated through the one-sample Kolmogorov-Smirnoff test. The comparisons of the means were performed by one-way ANOVA and the general linear model multi-variance by post-hoc analysis for pairwise comparisons. Correlations were evaluated by calculating the Spearman coefficient, and independent relationships were assessed via multiple regression analysis.

## Results

Table 1 shows the variables measured in PCOS patients. The statistical Kolmogorov-Smirnoff test proved a normal distribution for the measured parameters. The multiple regression analysis was applied to examine the relationship between obesity and testosterone, HOMA-IR levels, vitamin D levels of follicular fluid, and *VDR* gene expression on gramulosa cells. The results demonstrated that vitamin D levels of follicular fluid decreased in PCOS patients and over-weight individuals compared with non-PCOS and normal-weight patients. The results also revealed that 25 OH-D levels of follicular fluid were highly correlated with BMI (r=-0.51, P<0.01). In addition, HOMA-IR values were significantly higher in the women in PCOS/overweight and PCOS/normal weight than group those in non-PCOS/normal weight group (P<0.01). However, the difference in HOMA-IR values between the women in non-PCOS/overweight and non-PCOS/normal weight group was not significant (P=0.1, Table 1).

**Table 1:** Applied primer sequences for quantitative polymerase chain reaction (PCR)


Gene name	Primer sequence (5ˊ-3ˊ)	Accession number

VDR	F: ATACCAGGATTCAGAGACCTC	NM_000376.2
	R: TACTTGTAGTCTTGGTTGCCAC	
GAPDH	F: CGATGCGGCGGCGTTATTC	NM_002046.3
	R: TCTGTCAATCCTGTCCGTGTCC	


**Table 2:** Clinical and biochemical characteristics of studied women


	PCOS/Overweight n=20	PCOS/Normal weight n=18	Non-PCOS/Over weight n=19	Non-PCOS/Normal weight n=20

Follicle number	12.7 ± 3^b^	17.2 ± 4.2^c, d^	7.2 ± 1.2	9.1 ± 2
Age (Y)	29 ± 4.9	28.1 ± 4.1	28.1 ± 2.3	28.9 ± 4.2
BMI (kg/m^2^)	28.4 ± 2.7^a^	23 ± 1.9^d^	28.1 ±2.1^e^	22.5 ± 2
LH (IU/L)	7.4 ± 4.5^a, b^	9.2 ± 6.5^c, d^	4.8 ± 2.1^e^	6.5 ± 3
FSH (IU/L)	5.6 ± 1.7^b^	5.9 ± 1.7^c, d^	7.1 ± 2.5	7.2 ± 2.3
LH/FSH	1.4 ± 0.9^b^	1.6 ± 0.8^c, d^	0.9 ± 0.1	1.0 ± 0.2
Insulin (µmol/L)	24.7 ± 9.6^a^	11.6 ± 2.5^c^	19.6 ± 1.8^e^	13.7 ± 2.7
Glucose (mg/dl)	131.9 ± 30.5	111.96 ± 14.5^c^	120.46 ± 32^e^	95.4 ± 14.3
HOMA-IR	7.3 ± 1.4^a^	5.8 ± 0.9^c, d^	2.5 ± 1.1	2.3 ± 0.4
Testosterone (ng/ml)	1.9 ± 0.4	2.0 ± 0.7	1.7 ± 0.4	1.2 ± 0.5
Vitamin D (nmol/ml)	1.6 ± 0.9^a, b, c^	4.5 ± 1.7^d^	5.2 ± 1.8^e^	7.1 ± 1.3


Values are referred as mean ± SD. P<0.05 was considered statistically significant. ^a^; Significant differences between polycystic ovary syndrome (PCOS)/overweight and PCOS/normal weight, ^b^; Significant differences between PCOS/overweight and Non-PCOS/overweight, ^c^; Significant differences between PCOS/normal weight and Non-PCOS/normal weight, ^d^; Significant differences between PCOS/normal weight and Non-PCOS/overweight, ^e^; Significant differences between Non-PCOS/normal weight and Non-PCOS/overweight, BMI; Body mass index, LH; Luteinizing hormone, FSH; Follicle-stimulating hormone, and HOMA-IR; Homeostatic model assessment-insulin resistance.

Table 2 represents the correlation between age, BMI, and HOMA-IR and the number of follicles, serum testosterone levels, follicular fluid vitamin D levels, and *VDR* expression levels of granulosa cells. Furthermore, there was a considerable positive correlation between HOMA-IR and BMI (r=0.43, P<0.05). The results indicated that testosterone levels in PCOS/overweight patients were not substantially higher than those in non-PCOS/overweight patients (P=0.2). Similarly, there was not a significant correlation between BMI and testosterone (r=0.09, P>0.05, Table 3).

**Table 3:** Relationship between age‚ body mass index (BMI) and homeostatic model assessment-insulin resistance (HOMA-IR) with the studied variables


	Age	BMI	HOMA-IR

Follicle number	0.01	- 0.16	0.34
Testosterone	0.04	0.09	0.18
Vitamin D	- 0.12	- 0.51^**^	- 0.28
VDR	0.02	- 0.43^*^	- 0.41^*^


*; indicates significant difference from control at P<0.05 and **; Indicates significant difference from control at P<0.01.

**Fig.1:**
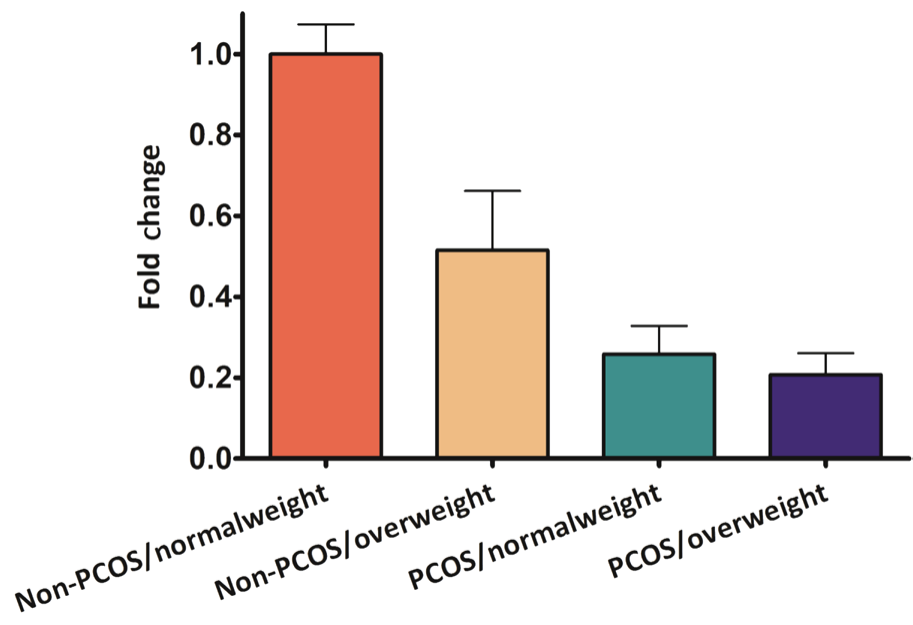
The fold change in gene expression patterns of VDR in polycystic ovary syndrome (PCOS)/overweight, PCOS/normal weight, non-PCOS/overweight, compared with non-PCOS/normal weight individuals. Expression levels are given as fold change compared with non-PCOS/normal weight samples. The error bars represent the 95% confidence intervals.

Quantitative RT-PCR results exhibited a lower *VDR* gene expression in PCOS patients compared to the control group (Fig .1).

The fold change in the expression of the target gene, *VDR*, was normalized to *GAPDH* in the PCOS/overweight, PCOS/normal weight, and non-PCOS/overweight groups. Afterwards, its expression in the control group, non-PCOS/normal weight, was analyzed. Melting curve analysis confirmed the specificity of the PCR (data not shown). A negative strong correlation was found between *VDR* expression levels and BMI (r=-0.43, P<0.05), using Spearman statistical test (Table 3). The gene expression data of VDR in granulosa cells were significantly lower (three times) in PCOS/overweight group in comparison with non-PCOS/normal weight (P<0.01). The level of *VDR* expression in PCOS/overweight group was similar to that of *VDR* expression in PCOS/normal weight group.

## Discussion

Two PCOS related complications are obesity and IR. Studies which show low vitamin D levels are associated with IR, and administration of vitamin D may ameliorate insulin sensitivity; however, the mechanisms of this effect are not clear (30). Moreover, studies demonstrate that insulin plays a significant role in the regulation of renal 1-a-hydroxylase activity and serum 1,25(OH) 2D3 levels in response to parathyroid hormone (PTH), while 1,25(OH) 2D3 is observed to act like a genomic stimulator of the insulin response in the control of glucose transport (31). Therefore, vitamin D may exert a positive effect on insulin action by stimulating the expression of insulin receptor, thus stimulating insulin responsiveness for glucose transport. Additionally, vitamin D responsive element is present in the promoter of the human *insulin* gene, and the transcription of *insulin* gene is activated by 1,25(OH) 2D3 (32). In accordance with the previous study, the current results confirmed that the majority of PCOS patients had vitamin D deficiency. There was a significant negative correlation between BMI increase and vitamin D levels of follicular fluid. It should be noted that the overweight PCOS individuals had lower vitamin D levels in their follicular fluid in comparison with other patients. Recent reports have indicated possible mechanisms for lower serum 25-OH-D3 associated with obesity. IR and obesity are also related to a reduction in growth hormone (GH) secretion in PCOS patients. This could be accounted for decreased levels of 1,25(OH) 2D3 because GH significantly increases renal 1-a-hydroxylase expression and, consequently, serum 1,25(OH) 2D3 concentrations (33). The findings revealed a negative correlation between vitamin D levels of follicular fluid and IR. Although this negative correlation was not substantial, its value level was imperative. Studies have also shown that obesity has been consistently associated with vitamin D deficiency, and this fact is supported in the present study by the negative correlation of weight and BMI with vitamin D level in follicular fluid (34). Hence, obesity can affect the decline of vitamin D concentrations in PCOS patients. On the other hand, *vitamin D receptors* are present in the majority of body cells, such as granulosa cells, and can influence the inhibition of cell proliferation and the induction of cell differentiation. Hence, vitamin D levels of follicular fluid and *vitamin D receptors* in granulosa cells may play an important role in proliferation and differentiation of granulosa and theca cells (35).

The relationship between allelic variation of VDR in pancreatic island and insulin secretion and glucose tolerance indicates a role for vitamin D in the pathogenesis of IR (36). Furthermore, vitamin D administration can improve insulin sensitivity and decrease insulin level (37). Accordingly, it is logical to state that in PCOS patients with increased BMI, vitamin D levels and *VDR* gene expression decrease, but IR increases (19). The findings indicated a significant negative correlation between *VDR* gene expression and HOMA-IR, which is in agreement with previous reports (38).

Based on the recent research, 25(OH) D levels are correlated with androgen levels in men, and one might speculate on an association of vitamin D with androgen. The underlying mechanisms, however, remain to be explored. The current study showed a negative correlation between vitamin D levels of follicular fluid and serum testosterone levels. Although this negative correlation is not significant, it is imperative. Vitamin D levels and intracellular calcium stores may regulate serum androgen levels of PCOS patients. As a result, vitamin D has a biologically plausible role in female reproduction, including the regulation of insulin secretion (39), androgen synthesis (40), proliferation of granulosa cells, and oocyte differentiation (41). In the previous studies, reduced vitamin D levels of serum in PCOS individuals were introduced as a possible factor affecting the reproductive disorders and metabolic disturbances of these patients (39). Overweight or obesity is one of the most important characteristics of PCOS individuals that may be employed as a powerful predictor of decreasing vitamin D levels. Previous studies have revealed the association of vitamin D status which improved IVF outcome. Women with higher levels of 25(OH) D in serum and follicular fluid were significantly more likely to achieve clinical pregnancy following IVF. On the other hand, high vitamin D levels were significantly associated with improved parameters of controlled ovarian hyper-stimulation (42). The present study revealed that the incidence of PCOS was associated with lower vitamin D levels of follicular fluid and decreased level of *VDR* gene expression in granulosa cells, which was more dominant in the PCOS patients with obesity.

## Conclusion

There are fundamental and significant differences in VDR gene expression in granulosa cells and vitamin D of follicular fluid in PCOS/overweight patients. Further investigations on larger sample populations are required to confirm that changes in the expression of *VDR* and vitamin D level of follicular fluid influence the development of different appearances of PCOS.
